# Hypoxiс postconditioning is an effective method of protection from severe hypoxia induced lipid peroxidation and neuronal apoptosis in rats

**DOI:** 10.1186/2193-1801-4-S1-P54

**Published:** 2015-06-12

**Authors:** Oleg Vetrovoy, Ekaterina Tyulkova, Elena Rybnikova

**Affiliations:** Pavlov Institute of Physiology Russian Academy of Sciences, St. Petersburg State University, Russian Federation, Russia

**Keywords:** Hypobaric hypoxia, hypoxic postconditioning, neuronal apoptosis, lipid peroxidation

Postconditioning (PostC) is an exposure of the damaged organism to extreme factors of the mild intensity to mobilize endogenous protective mechanisms. In our laboratory method of PostC using three daily trials of mild hypobaric hypoxia (MHH) was developed. It has been found that such method of the PostC effectively prevents degeneration of the hippocampal and neocortical neurons in rats, subjected to severe hypoxia (SH). Present study has been aimed at examination of the impact of oxidative processes in the development of the neuroprotection acquired in the course of hypoxic PostC during first three days of reoxygenation after SH in rats. The levels of thiobarbituric acid reactive substances (TBARS) and Schiff bases (SB) were used as markers of lipid peroxidation. In addition, the intensity of the apoptotic DNA fragmentation has been studied. During the three days after the SH a sustained increase of SB in the rat hippocampus was observed (700-1000% of the control value). After the first PostC episode the SB levels decreased to 150% of the baseline. Subsequently this parameter did not differ significantly from the control values. TBARS showed accumulation on the first day following the SH but afterwards its levels dropped to 40% of control and did not recover then to normal values. In the PostC animals, the levels of TBARS after each of three PostC episodes did not differ from the control values. These facts indicate that the PostC MHH balances the activity of pro- and antioxidant systems in vulnerable brain regions and promotes the effective utilization of components damaged by peroxidation. Fragments ladder typical for cells undergone apoptosis was obtained by the electrophoretic separation of the total DNA, extracted from a rat brain after one, two and three days after the SH. In the PostC group, the DNA fragmentation was revealed only after the first PostC episode, demonstrating antiapoptotic action PostC MHH.

